# Ameloblastoma With the Hybrid Desmoplastic and Plexiform Pattern: A Case Report

**DOI:** 10.7759/cureus.61686

**Published:** 2024-06-04

**Authors:** Ankita Chavhan, Aayushi Pakhale, Swati Patil, Alka Hande, Husna Tehzeeb, Sakshi Akolkar

**Affiliations:** 1 Oral Pathology and Microbiology, Sharad Pawar Dental College and Hospital, Datta Meghe Institute of Higher Education and Research, Wardha, IND

**Keywords:** hybrid ameloblastoma, desmoplastic ameloblastoma, plexiform ameloblastoma, odontogenic tumor, ameloblastoma

## Abstract

Ameloblastoma is an epithelial odontogenic tumor with a benign nature and demonstrates local aggressiveness. It frequently occurs between the third and fifth decades of life, showing significant gender predilection. While typically displaying a benign growth pattern, it tends to invade and sporadically metastasize locally. Ameloblastoma is predominantly found in the posterior regions. Periodic recur commonly follows insufficient treatment. Hence, conducting thorough identification of tumors and management is crucial to prevent relapse. Complications and improved prognosis are associated with meticulous surgical techniques, regular follow-up care, and early detection of recurrence. This study presented a report of a 19-year-old male with swelling in the left lower jaw, detailing its area of complaint, radiographic findings, histopathologic characteristics, and different treatment approaches. The uniqueness of the case is the hybrid histopathology of ameloblastoma composed of plexiform and desmoplastic variants.

## Introduction

Ameloblastoma is a type of tumor that develops from the remains of specific structures within the jaw, namely the dental lamina and enamel organ, both components of the odontogenic epithelium. This tumor is known for its aggressive behavior [1]. The term "ameloblastoma" was introduced by Churchill in 1933, while the first comprehensive account of this condition was provided by Falkson in 1879 [2]. Ameloblastomas have the potential to develop in various regions of the mandible or maxilla, although they are most commonly found in the mandibular molars and ramus, representing over 80% of all cases. The posterior region of the maxilla commonly occurs by the tumor [3]. Although ameloblastoma typically manifests between the third and fifth decades of life, it is noteworthy that this lesion can occur across all age groups, including children [4]. In 2022, the WHO classification delineated ameloblastoma into four variants: unicystic, conventional, metastasizing, and peripheral [5].

The typical manifestation of this condition often shows no symptoms and may be detected during routine X-ray scans or because of painless enlargement of the jaw [1]. Ameloblastoma may manifest as either a unilocular or multilocular radiolucency with a characteristic honeycomb or soap bubble-like appearance on radiological imaging. While a standard X-ray is adequate for detecting small lesions in the mandible, larger lesions in the maxilla or extensive ones necessitate the use of computed tomography (CT) and magnetic resonance imaging (MRI) scans to accurately assess the lesion's size and spread [6]. There are different histological patterns: follicular, acanthomatous, granular cell, desmoplastic, basal cell, and plexiform types [6]. The plexiform pattern is less aggressive and has a significantly lower recurrence rate [2]. The anterior portion of the jaw is where desmoplastic ameloblastoma is more commonly observed than other variations of the disease [7]. Various treatment approaches have been discussed, yet the consensus among most authors is that solid ameloblastoma requires aggressive and definitive management for a primary cure. Conservative methods like enucleation are linked with a notable recurrence rate [8].

## Case presentation

**Figure 1:**
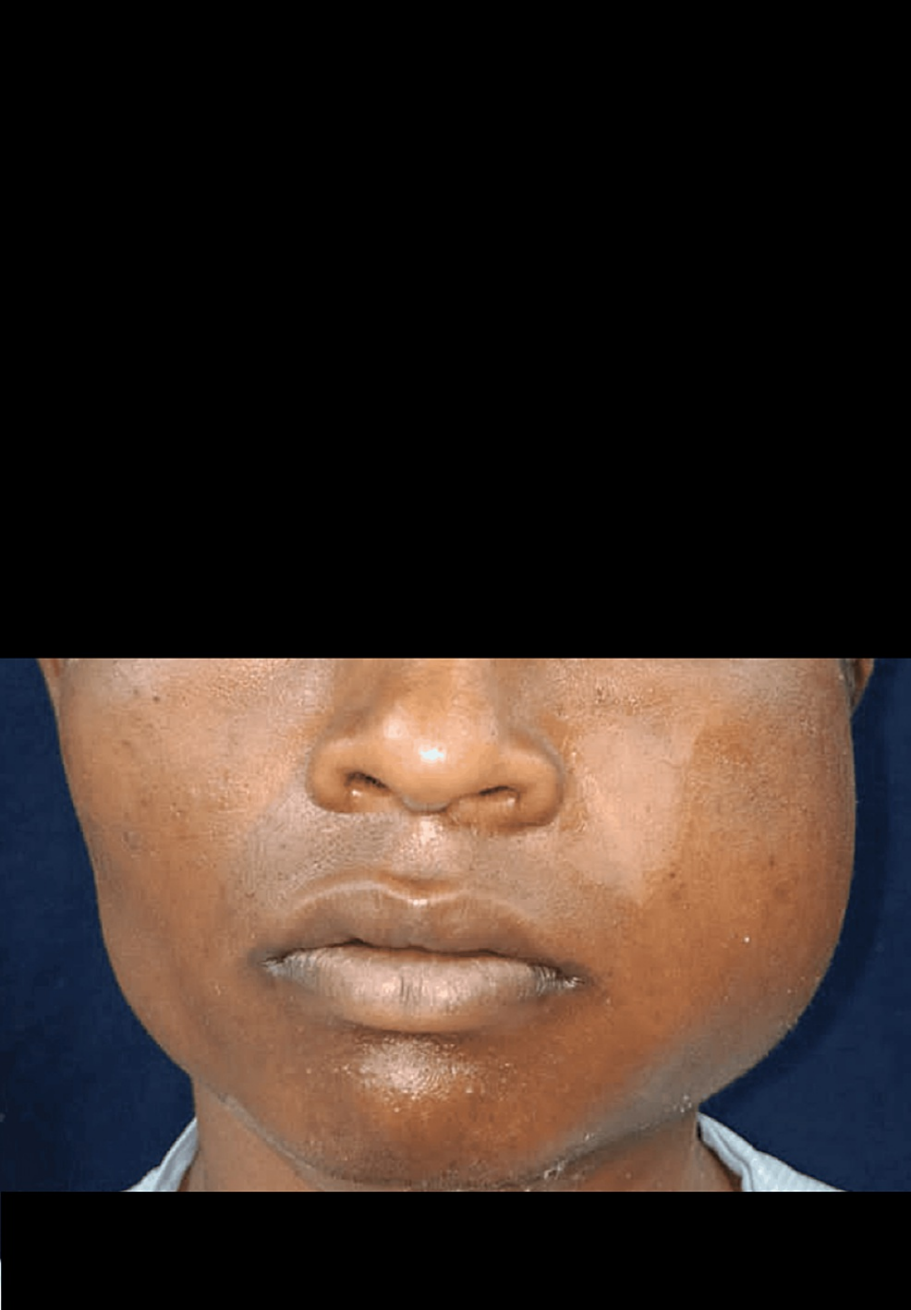
Clinically extraoral swelling is seen on the left side of the face

A 19-year-old male patient came with complaints of pain and swelling over the left side of the face for one month approximately. The patient was apparently alright one month back, but then he noticed swelling over the lower left posterior region of his jaw. There was no history associated with pain, which was dull aching, intermittent, and radiating toward the left ear and back of the neck. There were no relieving or aggravating factors. There was no history of trauma. The patient had a history of a change in saliva consistency from thin to thick and ropy, but there was no change in the quantity of saliva or difficulty in mastication. There was no difficulty in deglutition and no history of bleeding. There was pus discharge for approximately two days and a loss of appetite for about 10-15 days. There was no history of tooth exfoliation and nerve paresthesia. The patient first visited a private hospital where medication was prescribed. He then visited our hospital for further management. There was no past medical history, no history of blood transfusions, no past dental history, and no drug allergies. The patient had a habit of chewing kharra two to three times a day for the past two to three years. The musculoskeletal system was soft and non-tender. An extraoral examination showed facial asymmetry on the left side due to swelling, which extended anteroposteriorly from 1 cm posterior to the corner of the mouth to the posterior border of the ramus, and superior-inferiorly from the ala-tragus line to the inferior border of the mandible (Figure 1).

**Figure 2:**
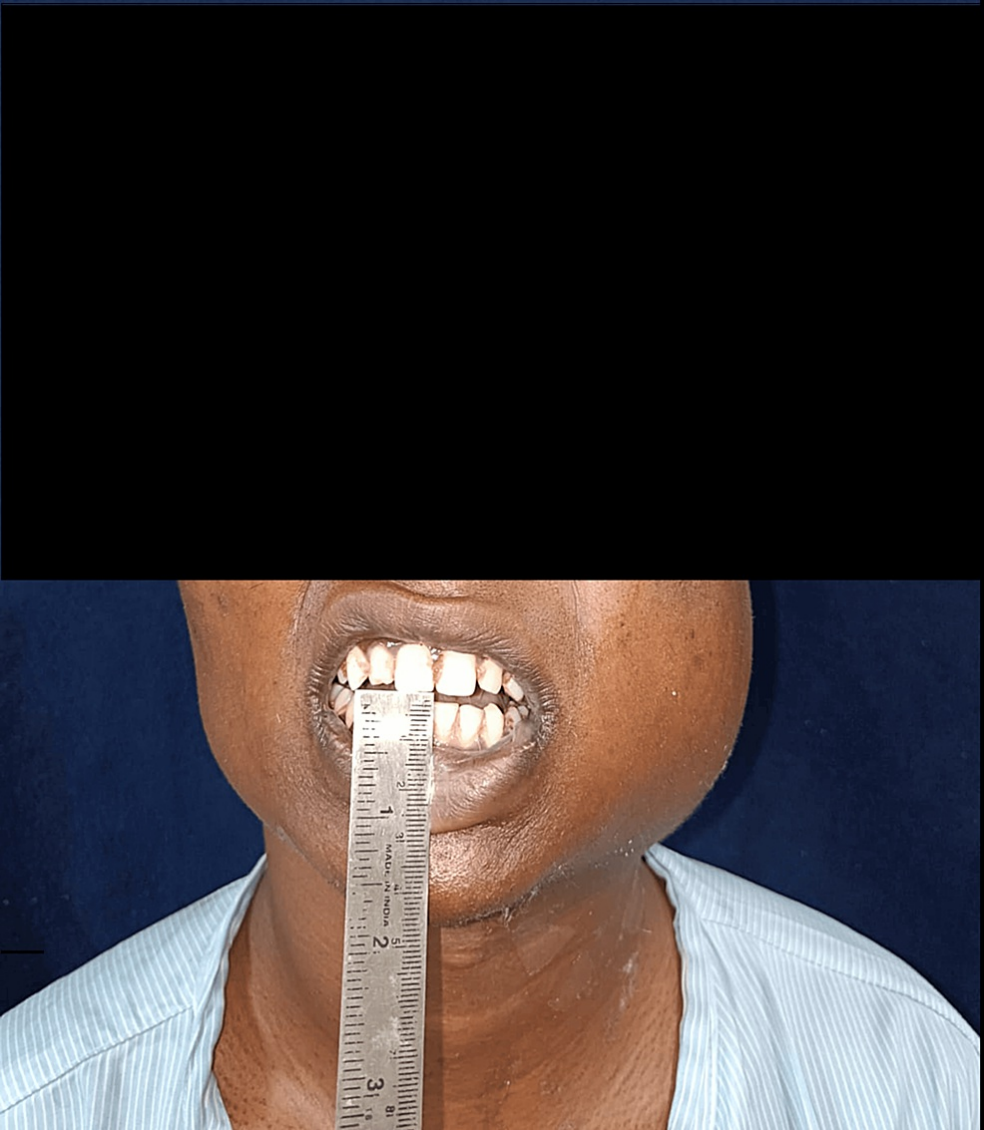
Intraoral clinical examination showing reduced mouth opening

The temporomandibular joint (TMJ) was bilaterally smooth and synchronous, but there was restricted jaw movement. Lips were competent. No abnormality was detected in the eyes/ear/nose, and clinically no lymph node was palpable. An intraoral examination revealed a reduced mouth opening of 4 mm. Teeth present included 11-17, 21-27, and 31-37, with 38 partially erupted and 41-47. A diffuse swelling, approximately 8 x 6 cm in size, was observed over the left mandibular region. Its shape was roughly oval, with diffuse margins, and its color matched that of the adjacent skin. On palpation, there was no tenderness and firm consistency (Figure 2).

**Figure 3:**
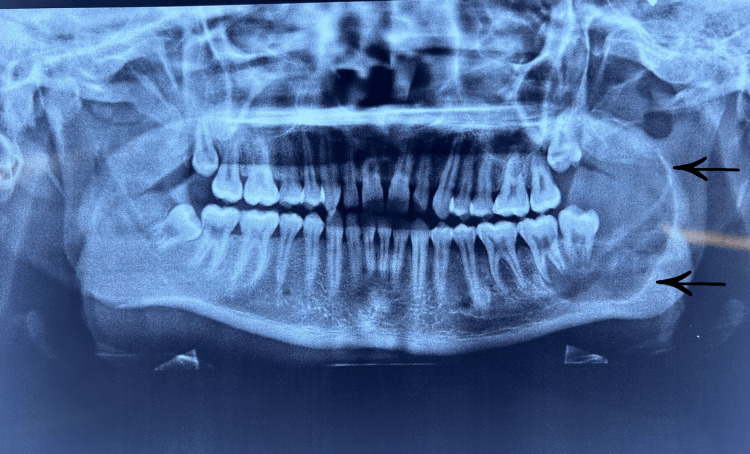
OPG showing unilocular radiolucency in the left ramus of the mandible associated with 37 and 38 OPG: orthopantomographic

An orthopantomographic (OPG) examination revealed a unilocular, corticated radiolucency which was clearly defined in the posterior mandible area (Figure 3).

**Figure 4:**
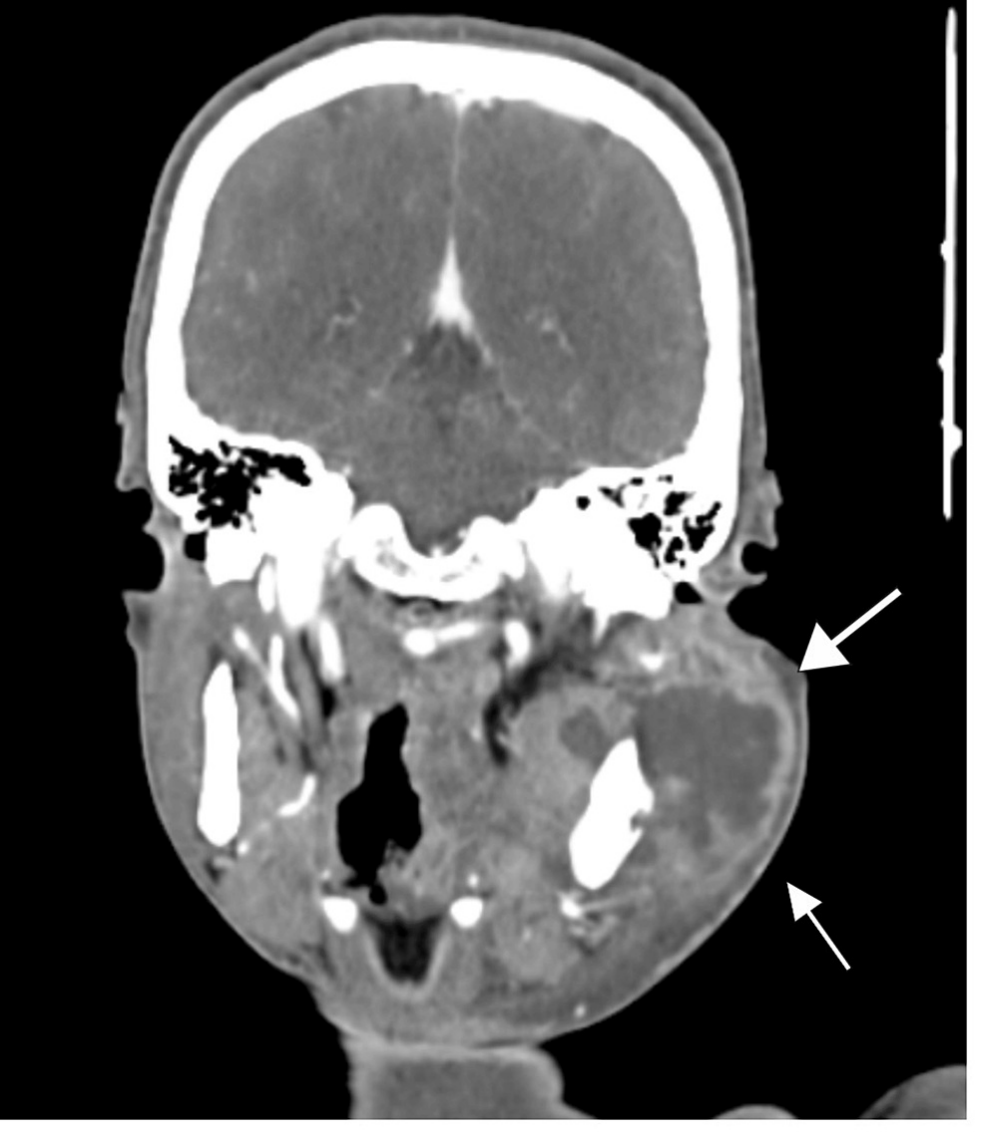
Heterogeneously enhancing irregular lesion seen on the left side of the mandible

**Figure 5:**
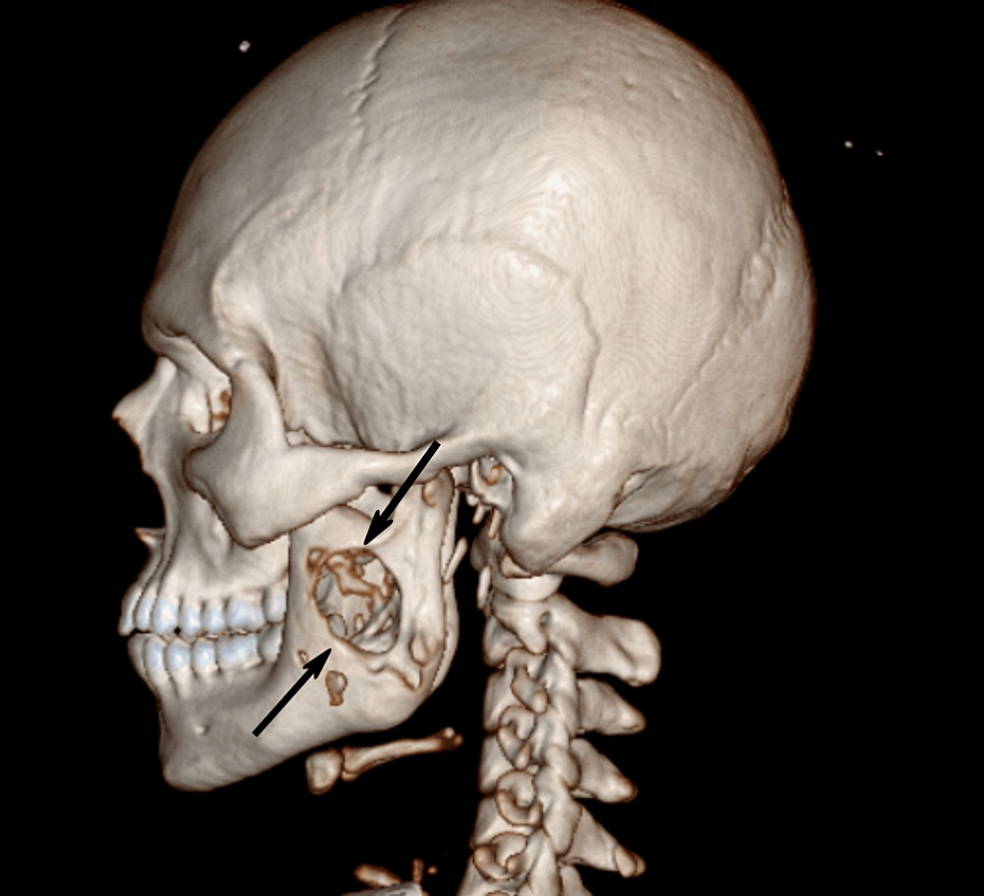
Lateral view of cone beam computed tomography (CBCT) showing a lesional defect in the left mandibular ramus area

The CT report showed that there is a heterogeneous enhancing irregular soft tissue density lesion with a surrounding peripherally enhancing collection, measuring approximately 7 x 5.5 x 7.8 cm, arising from the ramus of the left side of the mandible, causing its expansion and cortical breach, and extending to involve the soft tissue in the left masticator space and left pharyngeal mucosal space. The lesion had the following extension: laterally, involving the left masticator space with the involvement of the masseter muscle, reaching up to the subcutaneous plane. Medially, involving the medial pterygoid muscle and reaching up to the left pharyngeal mucosal space, with effacement of the oropharynx on the left side and loss of the fat plane with the base of the tongue on the left side. Superiorly, reaching up to the left TMJ with involvement of the retro maxillary space and infratemporal fossa. Inferiorly, reaching up to the left gingivobuccal sulcus, involving the left retromolar trigone. Posteriorly, abutting the left parotid gland, with heterogeneous enhancement noted in the left parotid (Figures 4-5).

**Figure 6:**
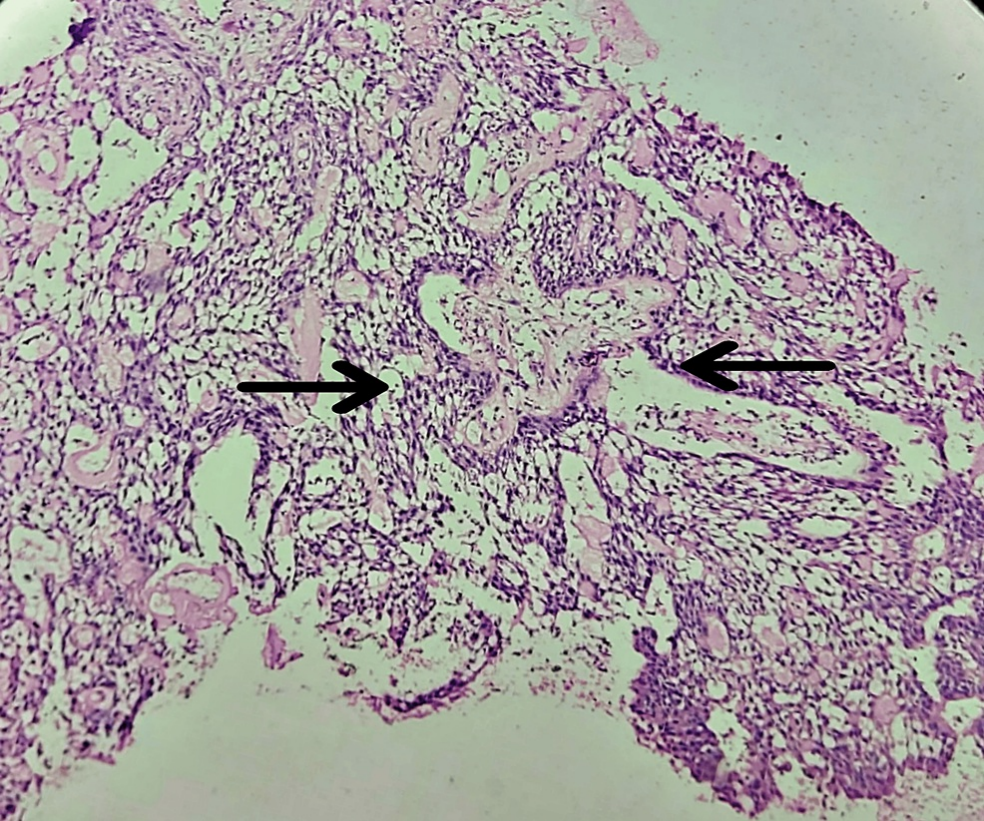
Microscopic picture showing strands of ameloblasts along with stellate reticulum (4x)

A local anesthetic-induced incisional biopsy was performed, and the specimen was received for histopathological examination, which showed lesional tissue composed of long strands and cords of tumor cells in an anastomosing pattern within loose areolar tissue stroma. The strands were bounded by columnar cells resembling ameloblasts, with adjacent cells resembling stellate reticulum, suggesting the odontogenic origin of the lesion. Therefore, it was diagnosed as plexiform ameloblastoma (Figure 6).

**Figure 7:**
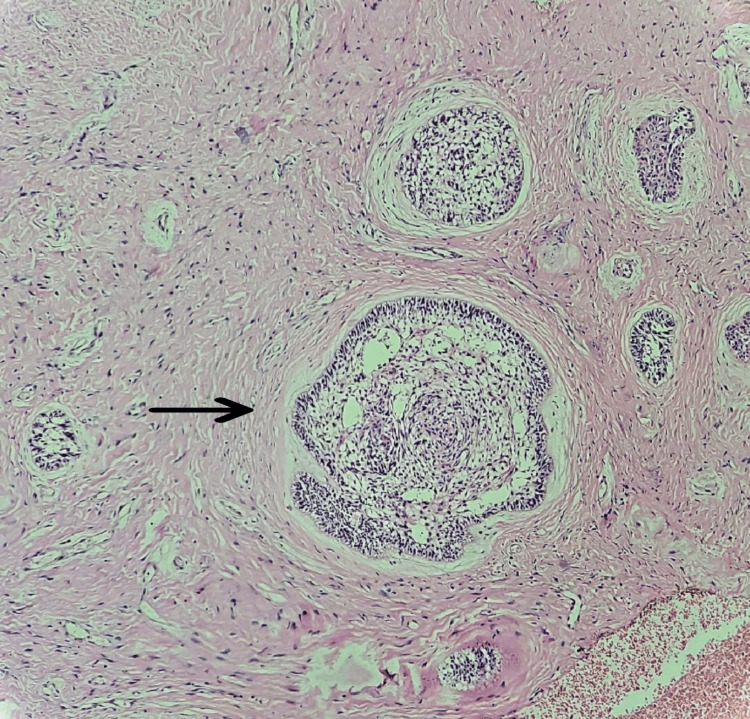
Low power view showing odontogenic follicles in connective tissue stroma (4x)

A wide local surgical excision of the tumor was done, with left segmental mandibulectomy from 36 to sub condylar region, followed by reconstruction with a recon plate under general anesthesia. Layer-wise closure was performed, and a pressure dressing was applied. The resected segment was received in the department for histopathologic examination. The histopathological examination showed tangled anastomosing cords and strands of odontogenic epithelium bounded by stellate reticulum-like cells appearance. The features were confirmatory for plexiform ameloblastoma (Figure 7).

**Figure 8:**
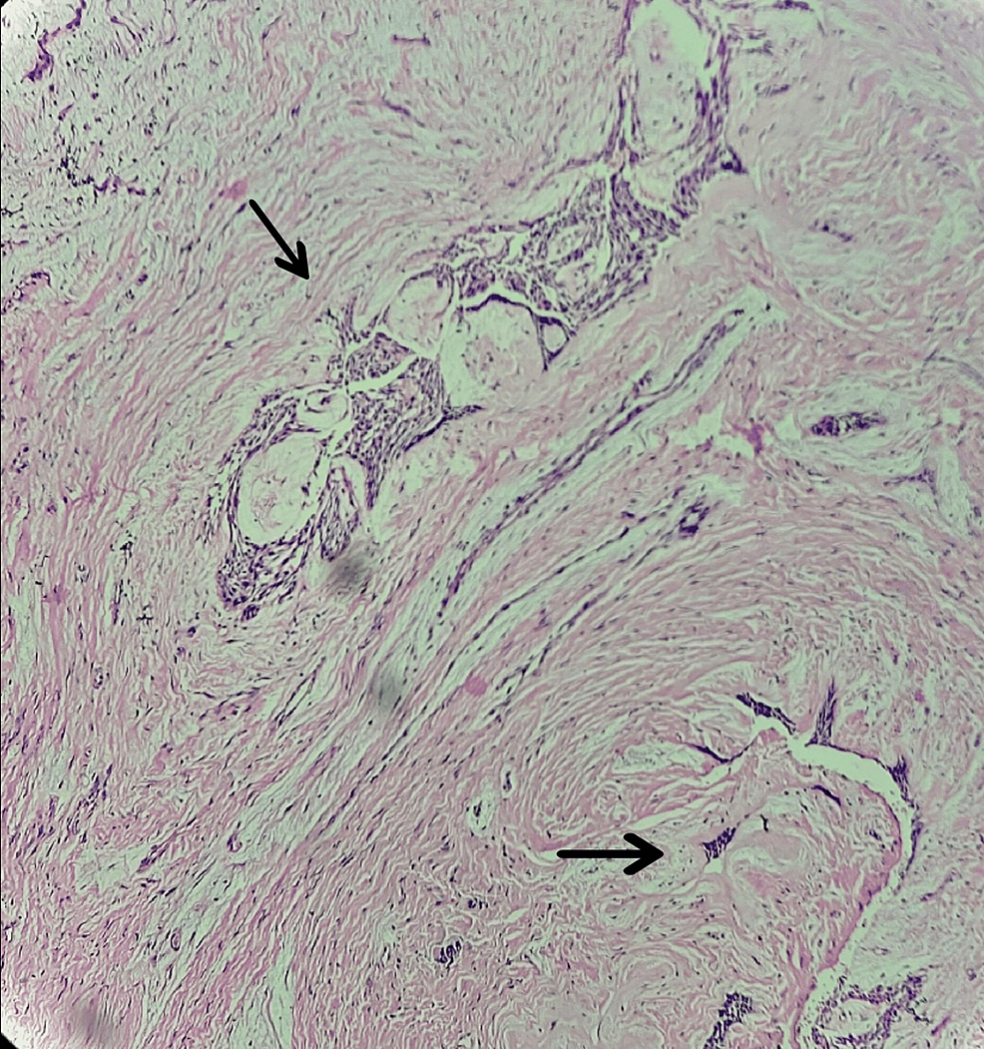
Under low magnification, a small odontogenic strand is seen surrounded by desmoplastic connective tissue (4x)

**Figure 9:**
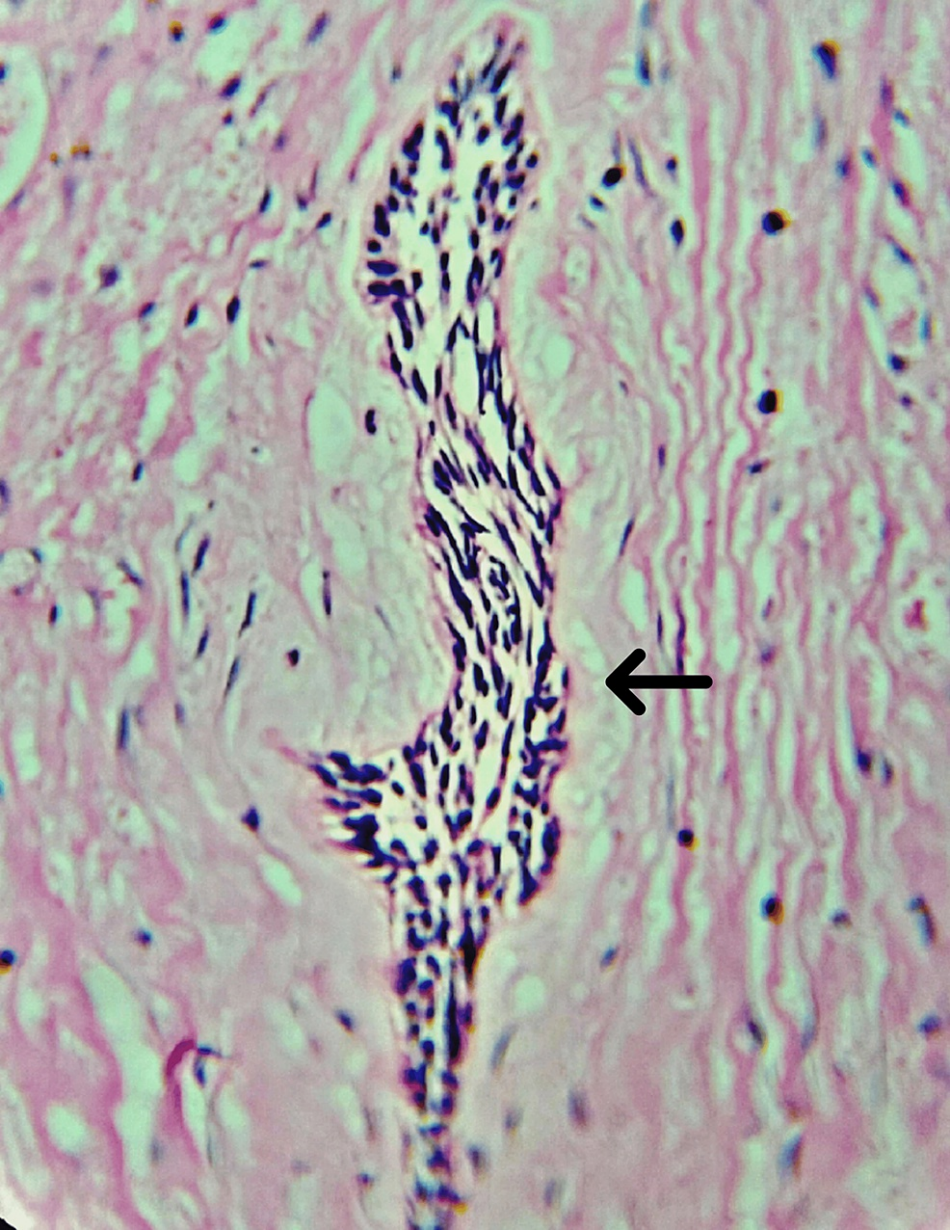
Under high magnification, odontogenic strands are compressed by the desmoplastic connective tissue (40x)

Some small to large tumor islands were seen and had central stellate reticulum-like cells, and peripheral cells were ameloblast-like cells forming odontogenic follicles. At places, tumor islands and cords were composed of columnar cells with hyperchromatic nuclei suggestive of odontogenic origin. The adjacent connective tissue was composed of a cellular fibrous stroma, with abundantly thick collagen fibers that were compressing the odontogenic epithelial islands from the periphery, resembling a desmoplastic variant (Figures 8-9).

**Figure 10:**
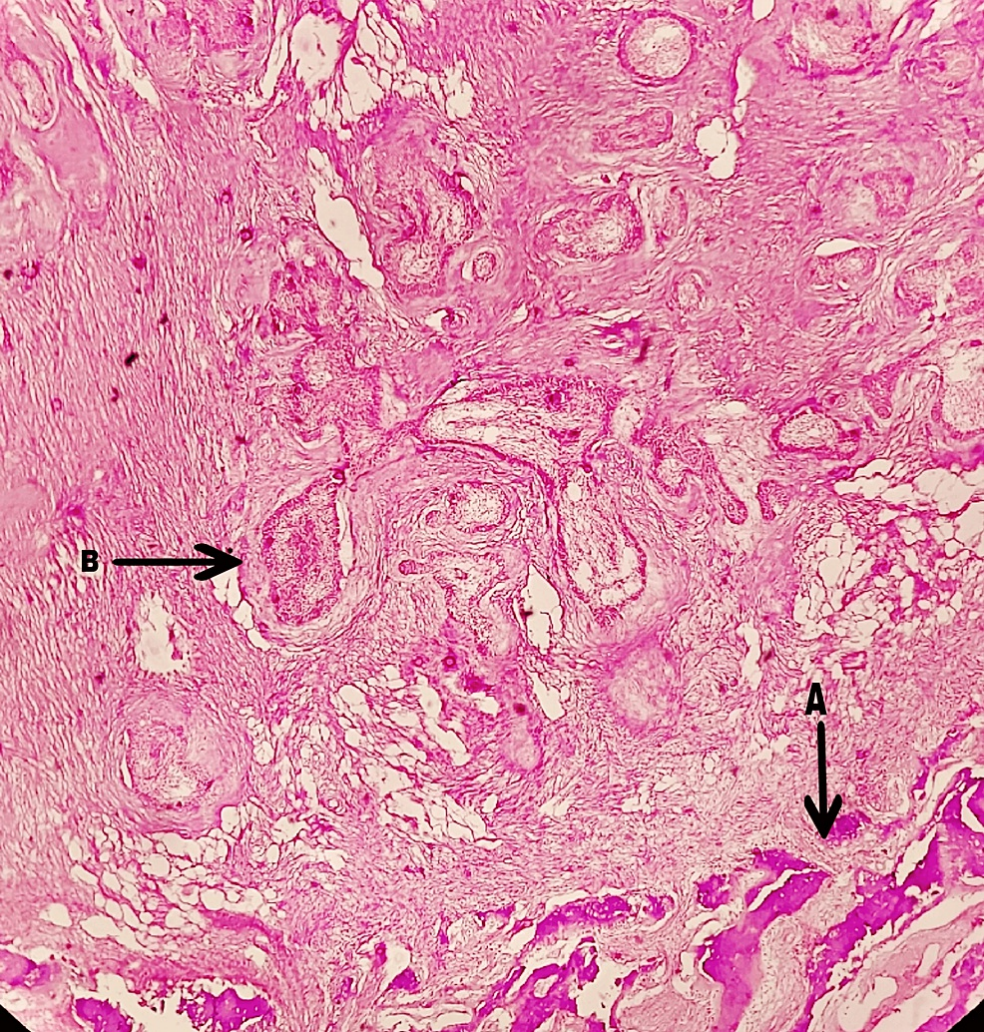
Decalcified H&E stained section showing invasion and destruction of bone by odontogenic island (4x) A: peripheral bony trabeculae; B: odontogenic islands causing bone destruction

The lesional tissue attached to bone was kept for decalcification in nitric acid, which revealed bony trabeculae of periosteum and central bone destruction by the lesional tissue composed of desmoplastic connective tissue and odontogenic islands (Figure 10). Thus, the histopathological diagnosis was concluded as plexiform ameloblastoma, with hybrid desmoplastic features.

## Discussion

Ameloblastoma holds the distinction of being the most prevalent clinically significant odontogenic tumor. Its frequency matches that of all other odontogenic tumors combined, with the exception of odontomas [1]. In 1963, Worth categorized ameloblastoma into four potential radiological presentations. In the first category, a cyst-like cavity is present with a partial absence of the wall, and faint septa can be observed within the lesion. In the second category, a striking resemblance to a spider's form may be evident in some instances, serving as a characteristic hallmark of ameloblastoma. The third pattern displays a multilocular cystic appearance, typically observed in the posterior region of the mandible and ramus. The fourth category is linked with the solid form of the tumor. It is characterized by replacing normal bone with a honeycomb-like appearance, where the cavities are relatively small and exhibit a fairly uniform size distribution [2,9]. In our case, the radiological presentation was present as multilocular radiolucency in the posterior region of the mandible, so it was categorized in the third category of Worth’s radiological manifestations.

The histological analysis plays a crucial role in cases of ameloblastoma. However, it's noted that numerous described histological patterns for ameloblastomas may not always hold clinical significance. Most ameloblastomas represent only one type of differentiation histopathologically, but certain types may display multiple histopathological differentiation patterns in lesional cells within the same lesion [1,10]. Our case report showed histopathological patterns of the hybrid type, which is a combination of plexiform and desmoplastic types. The plexiform type is the second most commonly occurring tumor about 28.2%, whereas follicular is the most frequently occurring type, about 32.5% of cases. Following closely is the acanthomatous subtype, comprising 12.1%. In contrast, the desmoplastic variant is exceptionally rare, with reported incidence rates ranging from 4% to 13% [3,11]. "Plexiform" describes the arrangement of branching islands of odontogenic epithelium, contrasting with the follicular pattern [6,12]. Desmoplastic ameloblastoma may be less extensive in size compared to other forms of ameloblastoma. However, if left untreated, it has the potential to spread widely and cause significant tissue damage, often necessitating thorough surgical removal [8,13]. Histologically, "hybrid lesions of ameloblastoma" include follicular or plexiform patterns coexisting with areas characteristic of desmoplastic ameloblastoma. The origin of these hybrid lesions is still uncertain, as it is difficult to hypothesize whether desmoplastic changes occurred secondarily in the stroma of a preexisting follicular or plexiform ameloblastoma, or whether areas of primary desmoplastic ameloblastoma convert into follicular or plexiform ameloblastoma. So such hybrid lesions are referred to as collision tumors [14,15]. The accepted approach to surgical treatment for ameloblastomas can be debated. The primary consideration often revolves around different histopathological variations, distinguishing between non-mural unicystic forms and solid multicystic types. While some authors suggest less aggressive treatments, such as enucleation or curettage of lesions, surgery with adequate bone margins is also effective for solid multicystic forms and intramural unicystic forms. The recommended treatment approaches for hybrid ameloblastoma are the same as those for solid ameloblastoma, which include curettage or total resection. However, small lesions can be easily enucleated in toto. According to the literature review, radical treatment options show a recurrence rate of approximately 8%, whereas conservative treatments have a higher recurrence rate of around 38% [5,6].

## Conclusions

An accurate and prompt diagnosis of the nature and extent of ameloblastoma is crucial, and this can only be achieved through a comprehensive microscopic examination of the lesion. We stress the importance of conducting a microscopic examination of all lesions resembling odontogenic lesions, before determining the treatment plan. Ensuring thorough radical resection of ameloblastomas is crucial to prevent subsequent complications and minimize the risk of recurrence. This particular case warrants attention due to its unusual appearance, potential aggressiveness, and misleading radiological characteristics, all of which increase the risk of misdiagnosis. Therefore, clinicians must remain vigilant to include desmoplastic ameloblastoma in the differential diagnosis of any lesion or growth presenting with a combination of radiolucent and radiopaque features, characterized by poorly defined borders, especially when the lesion is located in the maxilla or mandible.
